# Added Value of Comprehensive Program to Provide Universal Access to Care for Sputum Smear–Negative Drug-Resistant Tuberculosis, China

**DOI:** 10.3201/eid2507.181417

**Published:** 2019-07

**Authors:** Fei Huang, Susan van den Hof, Yan Qu, You Li, Hui Zhang, Lixia Wang, Miaomiao Sun, Wei Lu, Shuangyi Hou, Tianhua Zhang, Shitong Huan, Daniel P. Chin, Frank Cobelens

**Affiliations:** Chinese Center for Disease Control and Prevention, Beijing, China (F. Huang, Y. Qu, Y. Li, H. Zhang, L. Wang);; The Royal Netherlands Tuberculosis Foundation, The Hague, the Netherlands (S. van den Hof);; Academic Medical Center, Amsterdam, the Netherlands (S. van den Hof, F. Cobelens); PATH, Beijing (M. Sun);; Jiangsu Provincial Center for Disease Control and Prevention, Jiangsu, China (W. Lu);; Hubei Provincial Center for Disease Control and Prevention, Hubei, China (S. Hou);; Shaanxi Provincial Institute for Tuberculosis Control and Prevention, Shaanxi, China (T. Zhang);; Bill & Melinda Gates Foundation, Beijing (S. Huan);; Bill & Melinda Gates Foundation, Seattle, Washington, USA (D.P. Chin)

**Keywords:** tuberculosis, TB, tuberculosis and other mycobacteria, pulmonary TB, Mycobacterium tuberculosis, bacteria, drug-resistant tuberculosis, multidrug resistance, antimicrobial resistance, rifampin-resistant tuberculosis, line-probe assay, second-line therapy, comprehensive program, added value, universal access, care, respiratory infections, sputum smear, China

## Abstract

The increase in drug-resistant tuberculosis in China calls for scaling up rapid diagnosis. We evaluated introduction of rapid resistance testing by line-probe assay for all patients with a diagnosis of pulmonary tuberculosis in 2 prefectures in middle and eastern China. We analyzed sputum samples for smear-positive patients and cultures for smear-negative patients. We used a before–after comparison of baseline and intervention periods (12 months each) and analyzed data for 5,222 baseline period patients and 4,364 intervention period patients. The number of patients with rifampin resistance increased from 30 in the baseline period to 97 in the intervention period for smear-positive patients and from 0 to 13 for smear-negative patients, reflecting a low proportion of positive cultures (410/2,844, 14.4%). Expanding rapid testing for drug resistance for smear-positive patients resulted in a 3-fold increase in patients with diagnoses of rifampin-resistant tuberculosis. However, testing smear-negative patients had limited added value because of a low culture-positive rate.

Drug-resistant tuberculosis (TB) poses a major threat to TB control and elimination (*1*). China, where an estimated 73,000 patients showed development of rifampin-resistant TB (which requires longer and more toxic second-line treatment) during 2017, contains 13% of new cases of rifampin-resistant TB worldwide (*2*). However, of the 778,390 TB patients reported in 2017, only 14% were tested for drug resistance. Only 13,069 patients were reported to have rifampin-resistant TB, leaving >80% of cases undetected.

Recognizing the threat of rifampin-resistant TB, the Chinese Ministry of Health and the Bill & Melinda Gates Foundation have collaborated since 2009 to develop an improved TB control program to expand access to diagnosis, quality treatment, and affordable treatment for rifampin-resistant TB (*3*). In the first phase of the program during 2009–2011, pilot studies were conducted in 4 cities. In each city, 1 hospital was designated for diagnosis and treatment of rifampin-resistant TB and equipped with the Genechip line-probe assay (LPA; CapitaBio, http://www.capitalbiotech.com) for rapid molecular testing for isoniazid and rifampin resistance for all patients given a diagnosis of smear-positive pulmonary TB, rather than only those for whom rifampin-resistant TB was suspected (presumptive rifampin-resistant TB). In addition, collaborative mechanisms between the hospital, the local Center for Disease Control (CDC), and community health centers were set up to avoid loss of patients, specimens, and information as patients moved among these facilities. These pilot studies showed a 10-fold increased number of diagnoses of rifampin-resistant TB, a decrease in time from resistance testing to initiation of second-line treatment (by 90%), and an increased retention in treatment by 6 months, from 8% to 80% (*3*).

The Genechip LPA and the World Health Organization–endorsed GenoType MTBDRplus LPA (Bruker-Hain Lifesciences, https://www.hain-lifescience.de) were approved only for smear-positive sputum samples and culture isolates at the time of this study. A systematic review (3,451 samples in 4 datasets) of LPA performance for detecting TB showed a pooled sensitivity of 94% on smear-positive samples but only 44% on smear-negative samples (*4*). Because sputum cultures were not routinely performed in China, rapid resistance testing in the first phase of the program was limited to smear-positive TB patients.

In China, most (68% in 2017) reported patients with pulmonary TB are smear negative and given treatment without bacteriological confirmation and drug resistance testing. Therefore, China introduced a policy to scale up mycobacterial culture and rapid resistance testing. Its National TB Control Plan for 2016–2020, issued in February 2017, set targets of bacteriological confirmation for >50% of all reported patients with pulmonary TB and drug resistance screening for >95% of all patients with pulmonary TB at high risk for rifampin-resistant TB (*5*). In 2017, only 32% of TB cases in China were bacteriologically confirmed, 14% of pulmonary TB cases were tested for drug resistance, and 18% of estimated rifampin-resistant TB cases were diagnosed. The second phase of the program during 2012–2015 piloted a policy of adding sputum culture and LPA-based resistance testing of culture isolates to the diagnostic algorithm for smear-negative patients with pulmonary TB.

An alternative to culture and LPA testing might have been Xpert MTB/RIF (http://www.cepheid.com), an automated within-cartridge molecular assay, which tests for *Mycobacterium tuberculosis* and rifampin resistance and has been recommended by the World Health Organization as the primary diagnostic test for pulmonary TB where it can be afforded (*6*,*7*). Xpert can be used directly for smear-positive and smear-negative samples, and showed a pooled sensitivity similar to that for solid media culture (89%) when used as an initial test, but a lower sensitivity (67%) when used as an add-on test for smear-negative samples (*6*,*8*). Moreover, the sensitivity and specificity of Xpert MTB/RIF for detecting rifampin resistance are high and similar to that for LPAs (*9*), although it does not detect isoniazid resistance (*10*). However, because Xpert MTB/RIF has not yet been approved in China for case detection, it was not included as an alternative test in the study. Also, because the price for end-users is high (*11*), its scale-up as a first-line test in the TB and rifampin-resistant TB algorithm was considered less affordable than scaling up culture and LPAs.

In this study, we established the added value of expanding the diagnostic algorithm for diagnosis and treating bacteriologically confirmed pulmonary TB and rifampin-resistant TB. Specifically, we quantified the additional diagnostic yield, the number of case-patients needed to test to find 1 case of rifampin-resistant TB, the time to initiation of second-line treatment, and the number of patients lost in the diagnosis and treatment cascade for the culture-based algorithm.

## Methods

### Study Design and Population

The pilot studies combined innovative methods and tools with proven effectiveness, as evaluated in the first phase of the program, with health sector changes. These changes are being implemented by the Chinese National Health and Family Planning Commission. In addition to sputum culture and LPA-based resistance testing of culture isolates for smear-negative pulmonary TB, this second program phase introduced several other components. To reduce financial barriers to access to treatment, efforts were made to use health insurance reimbursement and other forms of social protection payment to reduce out-of-pocket costs for treatment of TB to 30% and for treatment of rifampin-resistant TB to 10%. As TB diagnosis and treatment tasks were being shifted from local CDCs to designated hospitals, a case-based payment mechanism was designed, which aimed for cost containment and standardized good clinical practice in care for patients with TB or rifampin-resistant TB (*12*,*13*).

We quantified the effect of implementation of the second phase of the program on the diagnosis of bacteriologically confirmed smear-negative pulmonary TB and rifampin-resistant TB in a before–after design. The program was designed to select 1 prefecture, respectively, from the eastern and middle regions of China as pilot study sites. Zhenjiang City (Jiangsu Province) and Yichang City (Hubei Province) were also selected based on good performance of features of the National TB Program in China (i.e., funding, laboratory capacity, and case detection). Prefectures consisted of the main municipality (city) and surrounding counties. The total population size in this pilot study was 6.9 million in 15 counties. Details of these prefectures have been described elsewhere (*14*). We assessed changes in numbers of patients given a diagnosis and compared a 1-year intervention period after implementation of the program (April 2014–March 2015) to a baseline period (January–December 2012). Both pilot study sites introduced all health sector changes.

### Diagnosis and Treatment

During the baseline period, presumptive TB patients (i.e., patients who had TB symptoms) underwent a chest radiograph and smear examination by Ziehl–Neelsen staining according to usual clinical routine. Clinical management was based on judgment of the clinician and national guidelines: clinical smear-negative TB diagnosis requires TB symptoms, chest radiograph abnormalities indicative of TB, and 3 negative sputum smear examinations. Both prefectures had experience with diagnosis and treatment of multidrug-resistant TB (MDR TB), which was defined as resistance to rifampin and isoniazid, through an earlier project supported by the Global Fund to Fight AIDS, Tuberculosis, and Malaria (GFATM) that included drug-resistance screening of patients at high risk for MDR TB by using sputum culture and phenotypic drug susceptibility testing (DST). However, after the GFATM project ended, the degree of implementation varied among sites.

During the intervention period, patients with presumptive pulmonary TB had a chest radiograph and smear examination. In addition, culture (1 sputum specimen in Löwenstein-Jensen medium) was used in county-level laboratories for smear-negative patients. Smear-positive samples and culture isolates were tested in city-level laboratories with Genechip or GenoType MTBDRplus assays. The Genechip test, a domestically developed diagnostic test, has a sensitivity of 87.6% and a specificity of 98.0% for rifampin resistance in this setting and a sensitivity of 80.3% and a specificity of 95.8% for isoniazid resistance (*15*). A systematic review of the GenoType assay showed a pooled sensitivity of 94.6% and a specificity of 98.2% for rifampin resistance, and a sensitivity of 83.4% and a specificity of 99.6% for isoniazid resistance (*9*). We compiled additional details of the diagnostic algorithm (Table 1).

**Table 1:** Diagnostic algorithm used during baseline and intervention period in study of added value of comprehensive program to provide universal access to care for sputum smear–negative drug-resistant TB, China*

Level	Baseline	Intervention
County	Light microscopy (Ziehl–Neelsen staining), smear examination, and chest radiograph for all presumptive TB patients	LED fluorescence microscopy (auramine staining), smear examination, and chest radiograph for all presumptive TB patients; culture for all patients with a diagnosis of smear-negative TB
City	Culture and phenotypic DST only for patients at high risk for MDR TB; culture for smear-positive patients in Zhenjiang	Line-probe assay (Genechip; CapitaBio, http://www.capitalbiotech.com) used in Yichang; GenoType (Hain Lifesciences, https://www.hain-lifescience.de/ en/company/contact.html) used in Zhenjiang for all bacteriologically confirmed pulmonary TB

*DST, drug susceptibility testing; LED, light-emitted diode; MDR TB, multidrug-resistant tuberculosis; TB, tuberculosis.

### Data Collection and Analysis

We extracted data for the baseline and the intervention periods from the routine electronic recording and reporting system for notification and management of TB patients (TBIMS) (*16*). This system contains data about demographic characteristics of patients, laboratory test results, TB diagnosis, treatment provided, and treatment outcomes. Patient data were entered at the clinic where the patient was registered for treatment. The system is maintained by the national-level CDC, which also provides supervision and data quality checks. Data collection and data capture format was similar for the 2 periods except for results of molecular tests, which were captured only for the intervention period. TBIMS data for diagnosis and treatment for patients with TB or rifampin-resistant TB were exported and merged by using unique identification numbers.

Pulmonary TB was defined as a diagnosis of pulmonary TB in the TBIMS. Bacteriologic confirmation was defined as TB confirmed by smear examination or culture. Rifampin resistance was determined by LPA or phenotypic DST on Löwenstein-Jensen medium.

We performed analyses by using Stata version 13 (https://www.stata.com). We compared numbers and proportions of patients given a diagnosis of any type of pulmonary TB, bacteriologically confirmed pulmonary TB, and rifampin-resistant TB between the baseline and intervention periods. We calculated reporting rates by dividing the number of observed cases by the population size, available by age and sex on district level, in 2012 for the baseline period and in 2014 for the intervention period. We used MDR TB patients in the baseline period as the comparison group for rifampin-resistant TB patients in the intervention period because rifampin-resistant TB patients and MDR TB patients were treated and managed the same way in the intervention period. Patients with TB resistant to isoniazid but not rifampin received the standard first-line treatment.

We tested distributions for categorical data by using 2-sided Fisher exact tests and for numerical data by using the nonparametric Wilcoxon rank test. We used logistic regression to determine patient characteristics associated with culture positivity for smear-negative pulmonary TB patients given a diagnosis during the intervention period, and characteristics associated with rifampin resistance for all bacteriologically confirmed pulmonary TB patients during the intervention period. Potential characteristics available and included in univariable and multivariable regression were age, sex, treatment history, disease severity, and migrant status. All statistical testing used 0.05 as the significance level.

## Results

A total of 4,553 pulmonary TB patients were reported during the intervention period and 5,269 during the baseline period. These findings reflected a 15% decrease in the annual reporting rate for all cases of pulmonary TB from 72.6 cases/100,000 persons to 61.7 cases/100,000 persons, which was consistent across the 2 prefectures (Yichang 9.2%, Zhenjiang 21.8%). After excluding 256 patients who had only pleural TB, we included 5,222 (99.1%) patients for the baseline period and 4,364 (95.8%) for the intervention period: overall, 1,808 (34.6%) smear-positive patients and 3,414 (65.4%) smear-negative patients during the baseline period, compared with 1,509 (34.6%) smear-positive patients and 2,805 (65.4%) smear-negative patients during the intervention period (Figure).

**Figure:**
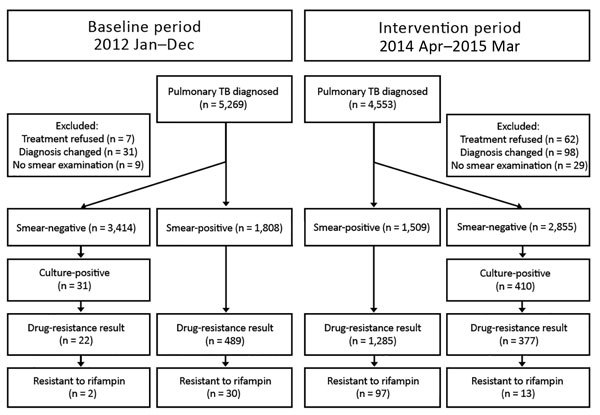
Flow diagram of TB patients given diagnoses at pilot prefectures in a baseline and intervention study of added value of a comprehensive program to provide universal access to care for sputum smear–negative drug-resistant TB, China. TB, tuberculosis.

### Culture Confirmation

During the baseline period 0.5% (12/2,258) of smear-negative patients were culture-confirmed in Yichang and 1.6% (19/1,156) in Zhenjiang. These proportions increased to 17.4% (358/2,055) in Yichang and 6.5% (52/800) in Zhenjiang, or 14.4% overall (95% CI 13.0%–15.7%), during the intervention period (p<0.001 for both prefectures) (Table 2). In Yichang, 5% of results were indeterminate, compared with 12% in Zhenjiang. The analysis of determinants of culture positivity among smear-negative patients showed strong effect modification by prefecture. Therefore, analysis was performed separately for each prefecture. In Yichang, the proportion culture-positive was independently associated with a history of previous TB treatment (adjusted odds ratio [aOR] 7.7, 95% CI 4.2–14.1), more severe disease (aOR 2.1, 95% CI 1.5–2.9), and being an internal migrant (aOR 3.1, 95% CI 2.2–4.1). In Zhenjiang, none of these determinants showed a significant association with culture positivity (Table 3).

**Table 2:** Drug resistance testing and results by prefecture and smear status in study of comprehensive program to provide universal access to care for sputum smear–negative drug-resistant tuberculosis, China*

Characteristic	Yichang, no. (%)			Zhenjiang, no. (%)	
	Baseline	Intervention		Baseline	Intervention
Smear-positive patients					
Patients given a diagnosis	1,177 (100)	949 (100)		631 (100)	560 (100)
Patients tested for drug resistance	36 (3.1)	863 (90.9)		470 (74.5)	518 (92.5)
Rapid resistance result available	36 (3.1)	828 (87.2)		453 (71.8)	457 (81.6)
Drug resistance test result					
No resistance to rifampin and isoniazid	24 (2.0)	715 (75.3)		405 (64.2)	389 (69.5)
Resistance to only isoniazid	3 (0.3)	46 (4.8)		26 (4.1)	38 (6.8)
Any rifampin resistance	9 (0.8)	67 (7.1)		21 (3.3)	30 (5.4)
MDR TB	6 (0.5)	30 (3.2)		14 (2.2)	18 (3.2)
Resistance to only rifampin	3 (0.3)	37 (3.9)		7 (1.1)	12 (2.1)
Smear-negative patients					
Patients given a diagnosis	2,258 (100)	2,055 (100)		1,156 (100)	800 (100)
Cultures performed	14 (0.6)	1,842 (89.6)		19 (1.6)	749 (93.6)
Cultures positive	12 (0.5)	358 (17.4)		19 (1.6)	52 (6.5)
Patients tested for drug resistance†	1 (<0.1)	352 (17.1)		22 (1.9)	53 (6.6)
Rapid resistance result available	1 (<0.1)	330 (16.1)		21 (1.8)	47 (5.9)
Drug resistance test result					
No resistance to rifampin and isoniazid	0	299 (14.5)		19 (1.6)	43 (5.4)
Resistance to only isoniazid	1 (<0.1)	19 (0.9)		0	3 (0.4)
Any rifampin resistance‡	0	12 (0.6)		2 (0.2)	1 (0.1)
MDR TB	0	10 (0.5)		2 (0.2)	1 (0.1)
Resistance to only rifampin	0	2 (0.1)		0	0

*Differences in numbers of patients tested and numbers with a test result reflect indeterminate test results. MDR TB, multidrug-resistant tuberculosis (i.e., resistant to rifampin and isoniazid); TB, tuberculosis. †Including 10 smear-negative patients who had no culture results and 24 smear-negative culture-negative patients for whom a line-probe assay was performed directly for sputum. ‡Including 2 smear-negative patients for whom a line-probe assay was performed directly for sputum.

**Table 3:** Patient characteristics associated with culture positivity for smear-negative TB patients, Yichang and Zhenjiang prefectures, China*

Characteristic	Yichang					Zhenjiang			
	Culture, no. (%)		Unadjusted OR (95% CI)	Adjusted OR (95% CI)		Culture, no. (%)		Unadjusted OR (95% CI)	Adjusted OR (95% CI)
	Negative	Positive				Negative	Positive		
Age, y									
0–39	401	102 (20.3)	Referent	Referent		166	12 (6.7)	Referent	Referent
40–59	565	129 (18.6)	0.9 (0.7–1.2)	0.9 (0.7–1.2)		215	14 (6.1)	0.9 (0.4–2.0)	1.0 (0.4–2.3)
>60	507	127 (20.0)	1.0 (0.7–1.3)	1.0 (0.8–1.4)		316	26 (7.6)	1.1 (0.6–2.3)	1.3 (0.6–2.8)
Sex									
M	1,001	260 (20.6)	Referent	Referent		514	38 (6.9)	Referent	Referent
F	472	98 (17.2)	0.8 (0.6–1.0)	0.8 (0.6–1.0)		183	14 (7.1)	1.0 (0.6–2.0)	1.1 (0.6–2.0)
TB treatment history									
New	1,455	325 (18.3)	Referent	Referent		604	42 (6.5)	Referent	Referent
Retreatment	18	33 (64.7)	**8.2 (4.6–14.8)**	**7.7 (4.2–14.1)**		93	10 (9.7)	1.5 (0.8–3.2)	1.6 (0.8–3.3)
Disease severity									
Not severe	1,313	284 (17.8)	Referent	Referent		645	48 (6.9)	Referent	Referent
Severe	160	74 (31.6)	**2.1 (1.6–2.9)**	**2.1 (1.5–2.9)**		52	4 (7.1)	1.0 (0.4–3.0)	1.0 (0.3–2.9)
Citizenship									
Local	1,326	267 (16.8)	Referent	Referent		523	34 (6.1)	Referent	Referent
Migrant	147	91 (38.2)	**3.1 (2.3–4.1)**	**3.1 (2.2–4.1)**		174	18 (9.4)	1.6 (0.9–2.9)	1.8 (0.9–3.3)
Total	1,473	358 (19.6)	NA	NA		697	52 (6.9)	NA	NA

*Values in bold indicate a statistically significant association (p<0.05). OR, odds ratio; NA, not applicable; TB, tuberculosis.

### Drug Resistance Testing

The proportion of smear-positive patients tested for drug resistance increased from 42.3% (899/2,126) to 83.0% (988/1,191; p<0.001) overall between the baseline and intervention periods, from 3.1% to 90.9% (p<0.001) for Yichang, and from 74.5% to 92.5% (p<0.001) for Zhenjiang (Table 2). In contrast, for smear-negative patients, the proportion tested for drug resistance increased between the baseline and intervention periods from 0.7% (23/3,414) to only 14.2% (405/2,855) (p<0.01); overall, from <0.1% (1/2,258) to 17.1% (352/2,055) (p<0.001) in Yichang and from 1.9% (22/1,156) to 6.6% (53/800) (p<0.001) in Zhenjiang.

### Drug Resistance Results

For smear-positive patients during the intervention period, rifampin resistance was detected in 7.1% (67/949; 95% CI 5.5–8.9) in Yichang and 5.4% (30/560; 95% CI 3.6–7.6) in Zhenjiang (Table 2). Overall, the number of smear-positive patients with rifampin resistance more than tripled from 30 during the baseline period to 97 during the intervention period.

For smear-negative patients during the intervention period, a drug resistance result was available for 16.1% of smear-negative patients in Yichang and for 5.9% of smear-negative patients in Zhenjiang. Rifampin resistance was detected in 13 patients with a smear-negative, culture-positive result: 3.5% of those tested with results, and 0.5% of all smear-negative patients. Two additional smear-negative patients who were culture negative showed rifampin resistance by LPA directly on sputum; thus, the physicians ordered the LPA directly on sputum, contrary to the guideline, to have the result as quickly as possible, because the patients had been considered at high risk for MDR TB.

The proportion of patients that showed rifampin resistance among those tested was significantly higher for smear-positive patients (97/1,381, 7.0%) than for smear-negative patients (13/405, 3.2%) (p = 0.005). Overall, the addition of culture and rapid resistance testing during the intervention period yielded 10.9% (13/119; 95% CI 5.9%–18.0%) additional diagnoses of rifampin-resistant TB and no significant difference between prefectures (p = 0.180). For isoniazid-monoresistant TB, the additional increase was 20.8% (22/106; 95% CI 13.5%–29.7%). A total of 13/2,591 patients were determined to have rifampin-resistant TB, and 22/2,591 were determined to have isoniazid-monoresistant TB.

### Second-Line Treatment

Of the 110 (including 13 smear-negative) rifampin-resistant patients with MDR TB given a diagnosis during the intervention period, 94 (85.5%) started second-line treatment, compared with 20 (60.6%) of 33 during the baseline period (p<0.001) (Table 4). The proportion that started treatment during the intervention period was 100% (13/13) for smear-negative patients and 83.5% (81/97) for smear-positive patients (p = 0.243).

**Table 4:** Smear results for patients who did and who did not start SLD treatment and outcomes for baseline and intervention periods in study of added value of comprehensive program to provide universal access to care for sputum smear–negative drug-resistant TB, China*

Characteristic	Baseline, no. (%)			Intervention, no. (%)	
	Smear-positive	Smear-negative		Smear-positive	Smear-negative
Initiated SLD treatment	18	2		83	11
Cure	9 (50.0)	1 (50)		14 (16.9)	0
Treatment completion	1 (5.6)	1 (50)		27 (32.5)	6 (54.5)
Death	2 (11.1)	0		9 (10.8)	1 (9.1)
Treatment failure	3 (16.7)	0		5 (6.0)†	1 (9.1)
Lost to follow-up	0	0		6 (7.2)	0
Stop treatment because of side effects	0	0		8 (9.6)	0
Other	3 (16.7)	0		14 (16.9)	3 (27.3)
Did not initiate SLD treatment	13	0		14	2 (18.2)
No regimen change, continued first-line treatment	11 (84.6)‡	0		2 (14.3)	0
Death	1 (7.7)	0		4 (28.6)	0
Lost to follow-up	0	0		3 (21.4)	0
Treatment refusal because of nonfinancial reason	0	0		1 (7.1)	1 (50.0)
Other	1 (7.7)	0		4 (28.6)	1 (50.0)
Total	31	2		97	13

*SLD, second-line drug; TB, tuberculosis. †Includes 10 patients with TB resistant to rifampin but not isoniazid, who were not eligible for SLD treatment during the baseline period. ‡Includes 2 patients given treatment for extensively drug-resistant tuberculosis.

For smear-positive patients, the median delay between presentation for diagnosis and initiation of second-line drug treatment decreased from 16 weeks during the baseline period to 16 days during the intervention period. For smear-negative patients, the median delay decreased from 21 weeks to 7 weeks. Among patients given second-line treatment, 12 (60.0%) of 20 patients during the baseline period and 47 (50.0%) of 94 patients during the intervention period had treatment success (cure or completion) as the outcome (p = 0.572). The proportion with treatment failure or death did not differ significantly between the intervention (16 patients, 17.0%) and baseline (5 patients, 25.0%) periods (p = 0.603). During the intervention period, 6 patients did not return for follow-up appointments and 8 stopped treatment because of side effects (total 14.9%), whereas neither finding was reported during the baseline period (p = 0.143). Three (9.1%) eligible patients during the baseline period and 16 (14.5%) patients during the intervention period were known not to have started second-line treatment (Table 4).

## Discussion

In this pilot study, expanding drug resistance testing for smear-positive TB patients suspected of having only MDR TB to all smear-positive TB patients resulted in a 3-fold increase in the number of patients identified having rifampin-resistant TB and a 5-fold increase in the number of patients given second-line treatment. This distinct increase was observed despite a reduction in numbers of pulmonary TB patients given a diagnosis at the 2 pilot study sites, which is consistent with decreased TB reporting in China (*2*). Introduction of rapid testing for rifampin and isoniazid resistance by LPA clearly was a decisive factor. The addition of mycobacterial culture, which enabled line-probe testing for smear-negative patients (i.e., those with low bacterial load in their sputum), yielded only 11% more cases of rifampin-resistant TB. This finding is a low yield given that smear-negative diagnosis accounted for 62% of all pulmonary TB cases; 199 cultures were required for each case of smear-negative rifampin-resistant TB detected. However, we detected 13 additional rifampin-resistant TB cases that would have been missed otherwise.

For smear-positive patients, the intervention decreased median delays until initiation of second-line treatment more than for smear-negative patients, reflecting the need for a culture isolate on which to perform LPA. However, this difference did not affect the proportion starting second-line treatment or treatment outcomes.

For smear-positive and smear-negative patients, we detected a similar absolute number of isolates resistant to isoniazid only as we detected rifampin-resistant isolates. At the time of this study, no differential regimens had been recommended yet for isoniazid-resistant TB, which could have led to improved regimens for 5%–7% of all patients given a diagnosis of pulmonary TB.

Addition of culture resulted in a low (14%) bacterial confirmation rate for smear-negative patients. This finding could reflect a high rate of false-negative cultures. False-negative sputum cultures are known to occur as a result of long sample transit times, harsh decontamination, or other shortcomings in laboratory procedures (*17*). The difference for the intervention period in the proportion of positive cultures between Yichang (19.4%) and Zhenjiang (6.9%) suggests that there were performance differences for the respective culture laboratories. An alternative explanation might be false-positive diagnosis of pulmonary TB based on minimal chest radiograph abnormalities and symptoms without any other test. This finding might imply overdiagnosis and overtreatment: a considerable proportion of these patients might not have had TB and therefore did not need anti-TB treatment. Conversely, the sensitivity of a single sputum culture on solid media, as performed in this pilot study, was not >90% (*6*), and studies have suggested that patients with typical chest radiograph abnormalities but repeatedly negative cultures are at high risk for development of culture-positive TB over the next 2 years (*18*). Irrespective of their cause, these low bacterial confirmation rates need to be taken into account when scaling up culture for resistance testing purposes in the current laboratory and clinical system in China.

Although the intervention of improved collaboration between hospitals and CDC, as well as improved health insurance, increased the number of patients receiving second-line treatment, a total of 14% of patients given a diagnosis of rifampin-resistant MDR TB did not start second-line treatment, half of them because they died or did not return for care before treatment could be initiated. In addition, treatment success of second-line treatment remained rather low (50%), and 25% of patients stopping treatment because of side effects or unknown reasons. This finding clearly calls for improvement of linkage into care and retention in treatment. In these pilot studies, patients with rifampin-resistant or MDR TB were given second-line drugs for 20–24 months; results for both categories might be improved by shortening second-line treatment to 9–12 months, as currently recommended for selected patients (*19*).

The new National TB Control Plan for China calls for capacity for culture, strain identification, phenotypic DST, and molecular resistance testing in all prefecture-level TB-designated hospitals, and for capacity for molecular resistance testing for 70%–80% of counties, districts, and cities throughout the country by 2020 (*5*). However, investments for scaling up this laboratory capacity will be substantial and need to include quality assurance, biosafety (for culture and DST), and prevention of cross-contamination (for PCR-based LPAs). Given the low additional yield of testing smear-negative pulmonary TB patients, scaling up use of Xpert, despite higher end-user prices, has a better cost–benefit ratio, and warrants formal analyses to establish cost-effectiveness and budget impacts by comparing various diagnostic algorithms and scale-up strategies (*20*).

Our study had limitations. First, the low bacterial confirmation rate for smear-negative patients might underestimate the effect of LPA for detection of rifampin-resistant TB, specifically in settings with higher bacterial confirmation rates. Second, both prefectures already had second-line treatment in place for patients given a diagnosis of MDR TB. Thus, these prefectures were not representative of the situation in most of China. This limitation implies that the increase in number of patients given a diagnosis of rifampin-resistant TB in our pilot study might have been less than in prefectures not supported through the GFATM, and the expected effect of new diagnostic interventions on case finding, for rifampin-resistant TB might have been underestimated. Third, patients who did not come to the county CDC (the so-called TB dispensary) were not included. Patients with presumptive TB at general hospitals should be referred to the TB dispensary; however, not all patients will be referred. Also, there would have been TB patients who did not come to general hospitals or TB dispensaries. It is not expected that self-referral and referral by general hospitals and village healthcare workers would have changed greatly from baseline to intervention periods. Fourth, we included only 2 prefectures that had different LPAs implemented. Therefore, we could not fully appreciate possible time effects on indicators, such as decreasing case reporting rates because of underlying epidemiologic changes.

Expansion of drug-resistance testing by rapid molecular assays to all pulmonary TB patients resulted in a 3-fold increase in numbers of patients given a diagnosis of rifampin-resistant MDR TB and who received appropriate treatment. Even so, testing smear-negative pulmonary TB patients had limited added value because of the low proportion of patients who had cultures positive for *M. tuberculosis*. Given the availability of alternative methods, such as Xpert, cost–benefit and budget impact analyses are warranted to determine if use of culture should be continued as part of the diagnostic algorithm to identify laboratory-confirmed TB cases. Despite several health system improvements, the linkage of care and retention of second-line TB treatment remained suboptimal.
